# Emerging roles for E3 ubiquitin ligases in neural development and disease

**DOI:** 10.3389/fcell.2025.1557653

**Published:** 2025-05-27

**Authors:** Maya Hale, Greg J. Bashaw

**Affiliations:** Department of Neuroscience, Perelman School of Medicine, University of Pennsylvania, Philadelphia, PA, United States

**Keywords:** neural differentiation, axon guidance, neural developmental disorders, E3 ubiquitin ligase, commissureless, Ndfip, slit

## Abstract

Neurodevelopment is an intricate process with highly regulated, overlapping stages including neuronal differentiation and axon guidance. Aberrations during these and other stages are tied to the etiology of neurodevelopmental disorders like Autism Spectrum Disorder, Angelman Syndrome, and X-linked Intellectual Disability. Ubiquitination is a dynamic and highly reversible post-translational modification conferred by E3 ubiquitin ligases. Recent discoveries have advanced the understanding of how substrate ubiquitination can guide protein localization, drive protein degradation, and alter protein post translational modifications. In this review, we highlight members of the RING and HECT E3 ligase families to discuss their novel roles in the molecular mechanisms regulating neurodevelopment. These findings are both instrumental for informing the future directions of neurodevelopmental research, and in expanding knowledge of intracellular mechanisms of protein trafficking. In addition, a deeper understanding of the molecular mechanisms of E3 ligase function in development promises to offer new insights into the pathogenesis of neurodevelopmental disorders.

## Introduction

Neurodevelopment begins with the specification of neural tissue and the differentiation of neural cells. Newborn neurons are then influenced by spatial and temporal hierarchies of extrinsic and intrinsic patterning signals that give rise to diverse neuronal populations. These neurons then migrate and extend axons and dendrites that contact target cells to form functional synapses. This process concludes with synapse maturation and the establishment of plastic circuits throughout the peripheral and central nervous systems (Alberts et al., 2002). These overlapping and tightly choreographed stages of neurodevelopment require extensive and highly dynamic changes in protein expression levels and localization. One versatile way to mediate these changes is through post-translational modifications of proteins.

Ubiquitination is an essential post-translational modification generated by the covalent linking of ubiquitin, a highly conserved 76 amino acid protein, to a protein target (Hershko and Ciechanover, 1998; Weissman, 2001; Akutsu et al., 2016). The process of ubiquitination is stepwise and requires three separate enzymes for the transfer of the ubiquitin onto a substrate. First the E1 enzyme (E1) activates the ubiquitin in an ATP-dependent reaction that creates a thioester-linked ubiquitin. Through this linkage, the E1 can then transfer the ubiquitin to the cysteine residue of an E2 enzyme (E2) (Haas et al., 1982; Kerscher et al., 2006). Then, the E2 coordinates with the E3 ligase to attach the ubiquitin group(s) through an isopeptide bond to substrate proteins (Johnson et al., 1995; Hershko and Ciechanover, 1998; Clague et al., 2015). The E3 ligase is also responsible for substrate recruitment, either through direct binding to the substrate (Cowan and Ciulli, 2022) or through binding to an adaptor protein (Mund and Pelham, 2009; Zheng and Shabek, 2017). All together there are around 600 E3 ligases in humans, which is orders of magnitude more than the one to two ubiquitin-modifying E1 enzymes and around 40 E2 enzymes encoded in the human genome (Schulman and Harper, 2009; Stewart et al., 2016; Jevtić et al., 2021). This vast diversity of E3 ligases and their myriad functions have generated sustained interest in understanding their roles in biological processes.

**FIGURE 1:**
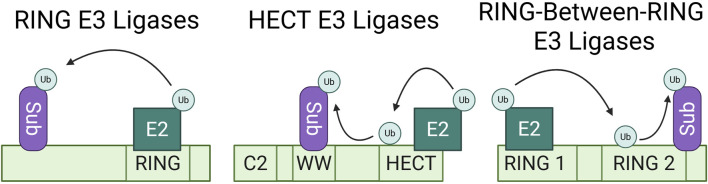
Most characterized E3 ubiquitin ligase families. Ubiquitination mechanisms of the RING, HECT, and RING-between-RING E3 ligase families. This simplified schematic shows direct E3 ligase-substrate binding, but each family can also employ one or more adaptors to bind substrates and bring them into proximity for ubiquitination. RING E3 ligases act as scaffolds for E2 enzymes, facilitating the direct transfer of ubiquitin to their proximal substrates. HECT family E3 ligases function as linkers between the E2 enzyme and their substrate. They temporarily accept the ubiquitin onto an available cysteine residue and the HECT domain later catalyzes the transfer of the ubiquitin onto the substrate. RING-between-RING E3 ligases share aspects of both RING and HECT catalytic mechanisms, wherein the RING1 domain binds the E2 enzyme and the RING2 domain temporarily accepts the ubiquitin, to then transfer the ubiquitin to the proximal substrate.

The three most characterized families of E3 ligases are distinguished by their catalytic mechanism of ubiquitin ligation (Figure 1). Really Interesting New Gene (RING) E3 ligases act as scaffolds for E2s by either forming a Zn^2+^ ion cross brace or through binding of the U-box and facilitating direct ubiquitin transfer to proximal substrates. Homologous to E6-AP C-terminus (HECT) family E3 ligases use a two-step process in which the HECT E3 first acts as a linker to accept the ubiquitin from the E2 onto a catalytic cysteine residue in the HECT domain and later catalyzes the transfer of the ubiquitin to the substrate lysine through a thioester bond (Kim et al., 2011; Metzger et al., 2014). In some cases, this requires a conformational change to expose the accepting cysteine. Lastly, RING-between-RING (RBR) family mechanism of catalysis shares elements of both the RING and HECT families; the RING domain binds the E2 similarly to the RING E3s, but this binding is in turn used to stabilize the transfer of the ubiquitin from the E2 to the catalytic domain of the RBR, which then transfers the ubiquitin to the substrate in an aminolysis reaction reminiscent of that of HECT E3 ligases (Wang et al., 2023). Each of these large families of E3s can be further stratified into subfamilies based on differences in substrate binding domains and catalytic domains. In addition to the major families, the recent discovery of the RING-Cysteine-Relay (Pao et al., 2018), ATP-dependent RZ finger (Ahel et al., 2021; Otten et al., 2021), and CRL-RBR-E3 (Horn-Ghetko et al., 2021) classes of E3 ligases have expanded understanding of ubiquitination mechanisms.

**FIGURE 2:**
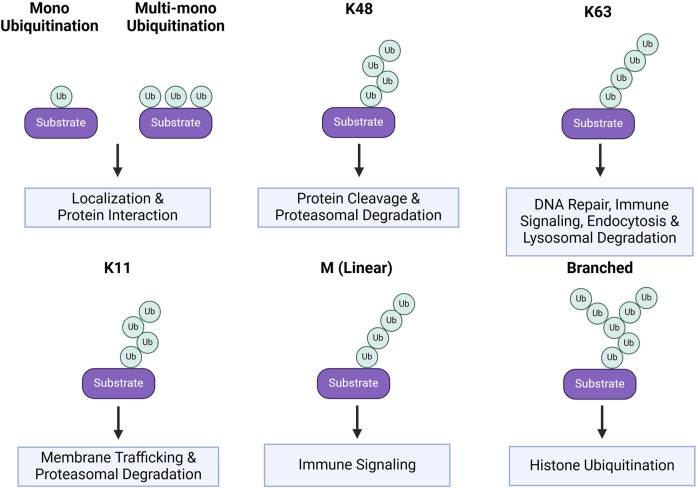
Ubiquitin linkages and substrate protein fates. Protein fates based on their ubiquitin linkage. Mono and multi-mono ubiquitination is when a single ubiquitin is conjugated to the substrate, rather than a chain. This form of ubiquitination generally alters the substrate localization or protein-protein interactions. These are also more transient post-translational modifications. K48, K63, K11, M, and Branched are all linkages in which chains of ubiquitin are conjugated onto the substrate. Chains linked at K48 result in protein cleavage and/or target the protein for proteasomal degradation. K63 ubiquitin chains have myriad effects including roles in DNA repair, immunity, endocytosis, and lysosomal degradation. K11 linkages result in changes in protein membrane trafficking and proteasomal degradation. M linkages occur at the N-terminal methionine of the protein and are associated with immune signaling. Lastly, branched ubiquitin chains are associated with histone ubiquitination.

The inducible and reversible transfer of ubiquitin canonically occurs at single or multiple available lysine residues, but can also occur at cysteine, serine, and threonine residues of a protein substrate, as well as non-proteinaceous lipids (Zheng and Shabek, 2017; Pao et al., 2018; McClellan et al., 2019; Mabbitt et al., 2020; Otten et al., 2021). These modifications can be classified as either mono or multi-mono ubiquitination, characterized by the conjugation of one molecule of ubiquitin (Dikic et al., 2009), or as poly-ubiquitination, characterized by the linkage of a polymerized ubiquitin chain(Figure 2). Other layers of complexity include the potential to ligate ubiquitin groups to N-terminal methionine (M1) residues, the selection of ubiquitin lysine residues (K6, K11, K27, K29, K33, K48, or K63) for chain elongation, and the subsequent types of homogeneous or heterogeneous poly-ubiquitin linkages (Komander and Rape, 2012; Swatek and Komander, 2016; Musaus et al., 2020).

Due to the various possible combinations of these ubiquitin modifications, the function of many linkages is still poorly understood. Of those that are better characterized, poly-ubiquitination at M1 is primarily implicated in immune signaling. Further, poly-ubiquitination at K63 is linked to a constellation of processes, including DNA damage repair, immune signaling, kinase activation, endocytosis, and entry into the endo-lysosomal pathway (Madiraju et al., 2022). Alternatively, poly-ubiquitination at K11 or K48 are associated with proteasomal degradation. Lastly, mono and multi-mono ubiquitination are associated with protein interactions, localization, and endocytosis (Suryadinata et al., 2014; Zinngrebe et al., 2014) (Figure 2). Generally, ubiquitin-induced endocytosis directs proteins to the endo-lysosomal degradation pathway, resulting in a range of fates from recycling to degradation in the lysosome. Ubiquitination is also a vital cue for the initiation of autophagy and binding of autophagy adaptors to proteins and organelles destined for degradation (Mizushima, 2024) (Figure 2). While the linkage-dependent outcomes for some proteins are well reported, the linkages conferred by each E3 ligase are not as well documented. For this reason, many E3 ligases are studied in the context of substrate interaction and downstream effects within a given signaling pathway. In this review, we highlight E3 ligases from the RING and HECT families with non-degradative and degradative functions in several neurodevelopmental processes and further discuss their implications in specific neurodevelopmental disorders (NDDs). Since the role of E3 ligases in synapse formation, function, and plasticity has been extensively studied and is the focus on several recent reviews (Widagdo et al., 2017; Mabb and Ehlers, 2018; Kawabe and Stegmüller, 2021; Mabb, 2021), our discussion will focus instead on the contribution of specific E3 ligase functions to neural differentiation, axon guidance, and dendrite morphogenesis. In the context of NDDs, we will highlight select instances where connections between E3 ligases and the regulation of specific substrate proteins have offered mechanistic insight into these disorders.

### Section 1: E3 ligases in neural specification

Specification of the neural plate from embryonic stem cells (ESCs) is coordinated by the spatiotemporal balance of secreted inhibitory factors and neural-promoting autocrine signaling (Gaspard and Vanderhaeghen, 2010). After neural plate formation, neurulation, and the specification of neural progenitor cells (NPCs), neural diversity is established through a series of lineage-dependent responses to spatiotemporal inputs. Morphogenic gradients and other external factors contribute spatial information for differentiation, and act as switches for cell-autonomous mechanisms.

**FIGURE 3:**
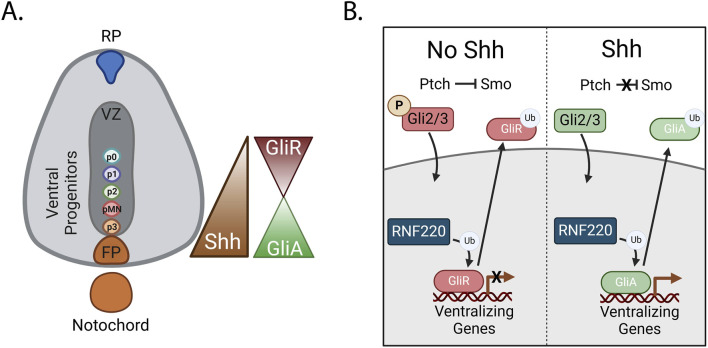
RNF220 regulates Shh signaling by ubiquitinating Gli proteins **(A)** Shh is secreted from the notochord and the floorplate. It then diffuses dorsally, creating a dorsal-ventral concentration gradient. Gli proteins are TFs expressed in the developing spinal cord. The repressive or activating function of Gli2 and Gli3 proteins is controlled by the expression levels of Shh. **(B)** Dorsally, where there are low levels of Shh, Gli2/3 proteins are phosphorylated, promoting their cleavage and resulting in a repressive function (GliR). Nuclear translocation and genomic binding of GliR results in the repression of ventralizing genes. Alternatively, when Shh is present, Gli2/3 are not phosphorylated and remain in an activating form (GliA). GliA translocation to the nucleus and genomic binding results in the transcription of ventralizing genes however, expression of the E3 ligase RNF220 ubiquitinates GliR and GliA, resulting in their transport out of the nucleus.

Sonic hedgehog (Shh) is an important morphogen in neural specification. After neurulation, Shh is expressed in both the notochord and the floorplate of the emerging spinal cord, producing a gradient along the dorsal-ventral axis, with Shh expression highest ventrally (Dessaud et al., 2008). Shh signaling and the dynamic activation and repression of its targets by Gli transcription factors (TFs), contributes to the expression of distinct and restricted patterns of ventralizing genes defining medial populations of ventral neuronal progenitors including the most ventral floorplate, p3, pMN, and least ventral, p2-p0 domains (Figure 3A). In later stages of spinal cord development, these progenitors give rise to interneurons, glial cells, and motor neurons (Lu et al., 2015; Ravanelli and Appel, 2015). Recent data implicates Ring Finger Protein 220 (RNF220), a highly conserved RING E3 ligase, in the tuning of Shh signaling and subsequent specification of ventral progenitor fates in the neural tube (Ma and Mao, 2022).

RNF220, a cytosolic protein, is expressed within the neural tube beginning at E8.5. RNF220 interacts with and ubiquitinates the Gli TFs (Ma et al., 2019). In mammals, three Gli TFs play key roles in the cellular response to the Shh gradient. Gli1, a direct target of Shh, functions exclusively as a transcriptional activator, contributing to a positive-feedback loop of Shh target gene expression. In the presence of Shh, Gli2/3 promote ventral fates by activating Shh target genes. On the other hand, in the absence of Shh, Gli2/3 are phosphorylated, enabling recognition for cleavage. Gli2/3 cleavage removes the Shh activating domain, resulting in repression of Shh target genes upon translocation of these TFs into the nucleus, and less ventralized cell fates (Hui et al., 1994; Ruiz i Altaba, 1998; Persson et al., 2002) (Figure 3B). In the absence of RNF220, mouse embryos display aberrant differentiation of ventral progenitor populations, with substantial increases in the p3 and p0 populations on the extreme ends of the Shh gradient and decreases in the p1 and p2 populations (Ma et al., 2019).

Interestingly, RNF220-mediated ubiquitination of both active and repressive forms of the Gli proteins results in decreased nuclear localization *in vitro* by improving the accessibility of a zinc-finger domain in the Gli proteins. This enables recruitment of CRM1 to drive nuclear export, ultimately modulating the expression of Shh target genes. The expansion of the p3 and p0 populations in RNF220 deficient embryos is likely due to an aberrant increase in activating Gli (GliA) TF binding in locations of high Shh availability and a reciprocal increase in repressive Gli (GliR) TF binding in more dorsal locations of low Shh availability (Ma et al., 2019) (Figure 3B).

Conditional knockout of RNF220 later in embryonic development also leads to alteration of the progenitor regions and their post-mitotic lineages in the hindbrain. By E12.5, the p0 domain and its daughter V0 interneurons remain expanded; however, loss of RNF220 exacerbates the subsequent decreases in V1 and V2 regions. Notably, while the pMN domain is still expanded, the p3 is also broadened. Given that Shh signaling is known to pattern both the embryonic spinal cord and the hindbrain, it is interesting that the alterations in progenitor domains due to the loss of RNF220 in the hindbrain are distinct from those in the spinal cord. In addition to the resulting differences in sMN/oligodendrocyte progenitors, there is also a significant increase of the serotonergic (5-HT) neuron population of the hindbrain, corresponding with p3 domain expansion. These findings may indicate a broader role for RNF220-mediated regulation in neuronal differentiation and psychiatric disorders associated with dysregulation of 5-HT circuitry (Wang et al., 2022).

In addition to regulating TF localization, E3 ligases and their adaptors also directly downregulate TF protein expression and play important roles in fine-tuning gene expression during neural specification in the cortex. For example, the Sox2 TF is expressed in neural stem cells (NSCs) and NPCs during early central nervous system (CNS) development, where it is required for NSC maintenance. *In vitro* models of ESCs also identified Sox2 as a TF for Shh, further linking it to known differentiation pathways (Favaro et al., 2009). *In ovo* inhibition of Sox2 leads to delamination of the ventricular zone and exit of the progenitors from the cell cycle, while constitutive expression of Sox2 inhibits neuronal differentiation and maintains progenitor characteristics through Oct3/4 (Graham et al., 2003; Masui et al., 2007). Accordingly, downregulation of Sox2 is crucial for the modulation of NPC fate and recent data implicates Cullin-RING finger ligase 4 (CRL4) complex in this process.

In one form of CRL4, Cullin4A (CUL4A) serves as a core scaffold for a RING finger binding protein, ROC1, that recruits E2 ligases. CUL4A also binds to one or more of the adaptor proteins, DDB1, DET1, and COP1, to interact with its target substrates and allow for their ubiquitination (Cheng et al., 2024). For example, Sox2 interacts with COP1 and is ubiquitinated by the CUL4A complex in NPCs. This ubiquitination and subsequent degradation of Sox2 increases over the course of development, resulting in neuronal differentiation of NPCs. Loss of DET1 and COP1 also abolishes the interaction between Cul4a and Sox2, thereby stabilizing Sox2 expression, further supporting the importance of CUL4A in Sox2 regulation. The novel Sox2 deubiquitinase, OTUD7B, is sufficient to prevent neuronal differentiation and maintain the NPC population, further reinforcing the importance of Sox2 ubiquitination and degradation by the CUL4 complex for timely NPC differentiation (Cui et al., 2018).

Interestingly, early studies of mouse ESC differentiation reported that Sox2 is ubiquitinated by WWP2, a HECT family E3 ligase, and subsequently degraded (Buckley et al., 2012; Fang et al., 2014); however, recent data report low levels of WWP2 expression in NPCs. This raises the question of how Sox2 is regulated in these NPCs. Additionally, Sox2 K119 mono-methylation causes a conformational change that facilitates its ubiquitination by WWP2, but CUL4 complex-mediated ubiquitination is independent of Sox2 K119 mono-methylation, indicating that despite regulating the same protein, WWP2 and the CUL4A complex likely utilize a different Sox2 ubiquitination site. This could be due to differences in substrate recognition and/or enzymatic activity inherent to RING E3s and HECT family E3s. This difference in binding combined with low levels of WWP2 expression in NPCs could be evidence of a cell-specific Sox2 mechanism of ubiquitination and regulation found in NPCs, but not in the ESC pool (Cui et al., 2018). Data revealing critical roles for RNF220 in Shh signaling in the spinal cord and hindbrain, and CUL4A in Sox2 regulation in the cortex, exemplify the importance of E3 ligases in neural differentiation.

### Section 2: E3 ligases in axon guidance

Newly differentiated neurons project their axons toward synaptic targets to form functional circuits. Guidance of these axons is mediated by the spatiotemporal regulation of attractant and repellant receptors on the membrane of the growth cone, a highly motile structure at their axon terminal (Evans and Bashaw, 2010). Binding of secreted and membrane-tethered axon guidance cues to these trans-membrane receptors leads to downstream signaling. This binding which remodels the growth cone plasma membrane and cytoskeleton to allow for directional growth responses (Chédotal, 2019). Ligand binding frequently leads to receptor internalization and receptor cleavage events that are intimately associated with receptor regulation and signaling. Endocytosis of receptors alters growth cone responsiveness by tuning the surface levels of receptors and can also play a vital role in initiating downstream signaling (O’Donnell et al., 2009). Receptor cleavage can regulate local signaling to the cytoskeleton and allow for nuclear translocation of intracellular domains (ICD) fragments that can regulate transcription. The ability of receptor ICDs to regulate transcription adds another layer of regulation to the process of axon guidance and suggests that guidance receptor signaling may also control additional aspects of neuronal maturation and function (Zang et al., 2021). Cytoskeletal rearrangement, endocytosis, and cleavage all facilitate the dynamic gradient- and receptor-dependent directionality of growth cone extension (Evans and Bashaw, 2010; Zang et al., 2021). In this section, we will discuss some of the roles of E3 ligases in the process of axon guidance with a particular emphasis on recent studies of Netrin-dependent axon attraction and Slit-dependent axon repulsion.

#### Netrin-mediated attraction

During axon guidance, Netrin is secreted from the floor plate and ventricular zone in the spinal cord, and in multiple cortical and subcortical regions (Wu et al., 2019). Netrin binding to *Drosophila* Frazzled (Fra) or vertebrate deleted in colorectal cancer (DCC) induces canonical chemoattractant signaling resulting in cytoskeletal rearrangement (Harris et al., 1996; Moore et al., 2007). Some downstream targets of Netrin-Fra/DCC signaling include the WAVE regulatory complex (WRC) which activates Arp2/3 to promote branched actin network assembly and Mena/VASP family of actin-regulatory protein which prevent actin capping and facilitate the formation of long unbranched actin filaments (Drees and Gertler, 2008; Chaudhari et al., 2024). In the context of Netrin-DCC signaling, Ena interacts with the barbed end of F-actin, increasing protrusion and extension of filopodia for growth cone attraction (Lebrand et al., 2004).

**FIGURE 4:**
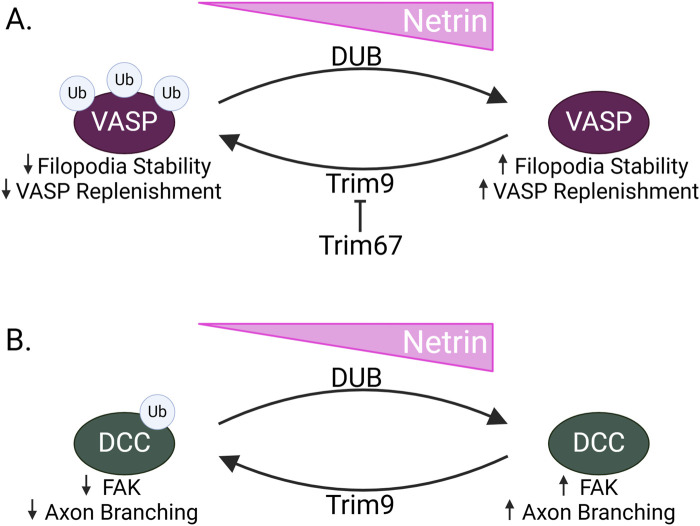
TRIM9 and TRIM67 in Netrin signaling **(A)** When Netrin expression is low in neurons during axon guidance, the RING E3 ligase Trim9 ubiquitinates VASP. This results in decreased filopodial stability and decreased replenishment of VASP within filopodia. Conversely, when Netrin levels are high, VASP is deubiquitinated by a deubiquitinating enzyme, resulting in increased filopodial stability and VASP replenishment. Trim67, another RING E3 ligase, inhibits Trim9, acting as a switch to allow for altered filopodial dynamics in response to Netrin. **(B)** Trim9 also ubiquitinates DCC when Netrin is low. This decreases FAK binding and prevents FAK-induced axon branching. In the presence of Netrin, DCC is deubiquitinated, allowing for increased FAK signaling and increased axon branching.

Recent data links two RING family E3 ligases, Trim9 and Trim67, with the regulation of Mena and filopodial extension (Figure 4) (Menon et al., 2015; Plooster et al., 2017; Boyer et al., 2018; Boyer et al., 2020). Trim9 is expressed in the growth cone of cortical neurons during embryonic mouse development and endogenous Trim9 interacts with Mena, VASP, and EVL. *In vitro*, Trim9-Mena/VASP interaction leads to VASP ubiquitination. Notably, VASP ubiquitination does not decrease VASP protein expression but instead alters VASP protein localization at filopodial tips. Interestingly, a ubiquitin group can be ligated to three separate lysines of VASP. This suggests that VASP could be multi-monoubiquitinated, a linkage associated with altered protein localization and interaction dynamics, further supporting that Trim9 ubiquitination regulates VASP outside of a degradative pathway (Dikic et al., 2009) (Figure 4). Trim9 ubiquitination of VASP may be important for regulating filopodial stability as *in vitro* knockout of *TRIM9* increases growth cone area and increases the duration of filopodial extension, and the number of filopodia. This effect requires the presence of the VASP protein, as well as the Trim9 domains that are responsible for interaction with VASP (Menon et al., 2015).

Despite the propensity of Trim9^−/−^ primary neurons to grow more filopodia, addition of Netrin does not potentiate this increase. Interestingly, switching between the ubiquitinated and un-ubiquitinated VASP may be required for Netrin response as there is no *in vitro* response to Netrin in the presence of either non-ubiquitinatable VASP mutants or in conditions preventing VASP deubiquitination. This supports a model in which Trim9 ubiquitinates VASP, altering its localization at filopodial tips. It is also possible that recruitment of Trim9 to filopodia by Mena/VASP/EVL facilitates the ubiquitination of many VASP proteins, maintaining a less-stable and more motile state of the filopodia; however, upon Netrin stimulation, VASP is deubiquitinated, allowing for increased filopodial stability and Netrin-induced attraction (Menon et al., 2015) (Figure 4A).

Trim9 also plays a role in Netrin signaling though its ubiquitination of DCC in neurons. Akin to the ubiquitination of VASP, Trim9-mediated DCC ubiquitination in primary cortical neurons does not decrease protein expression but appears to promote DCC multimerization and aggregation in the absence of Netrin (Menon et al., 2015). This is significant because the DCC crystal structure and DCC-Netrin binding affinity suggest that the cytosolic domain of DCC must dimerize for Netrin-induced attraction (Finci et al., 2014).

Within the cytoplasmic tail of DCC, there are FAK and SFK binding sites with two of the potential ubiquitin-binding lysines flanking the FAK binding site. These FAK and SFK binding sites recruit nonreceptor tyrosine kinases to DCC and are implicated in axon outgrowth in response to Netrin (Li et al., 2004; Ren et al., 2004). Both the loss of *Trim9* and mutation of the ubiquitin-accepting lysines result in increased interaction with and activation of FAK, suggesting that DCC ubiquitination sterically hinders binding of FAK, preventing downstream FAK/SFK signaling. In accordance with increased Trim9 substrate ubiquitination in the absence of Netrin, the loss of *Trim9* abolishes the Netrin response. The *in vivo* importance of Trim9 in the regulation of FAK-induced axon branching was investigated in the mouse corpus callosum, where loss of *Trim9* increased branching, in line with the purported effect of decreased DCC ubiquitination and subsequent increases in FAK signaling. In line with this, the branching phenotype is rescued by removing FAK (Figure 4B). Together, this suggests that Trim9 is not only impacting filopodial stability but may also inhibit axon branching by ubiquitinating DCC (Plooster et al., 2017).

Trim67 is also connected to filopodial stability through its regulation of VASP activity. Similarly to Trim9, Trim67 is highly expressed in the embryonic cortex and localizes to the growth cone (Boyer et al., 2018). It also colocalizes and interacts with VASP at growth cone filopodia *in vitro* and knockout of *Trim67* increases growth cone area; however, the direct comparisons to Trim9 end here. In contrast, Trim67 decreases VASP ubiquitination, through an undefined mechanism. As an E3 ligase, it is possible that Trim67 ubiquitinates Trim9, promoting its degradation and preventing VASP ubiquitination. Alternatively, it could downregulate a protein within the deubiquitination pathway, promoting deubiquitinase activity that antagonizes VASP ubiquitination. Additionally, Trim67 affects filopodial dynamics like protrusion and retraction in primary cortical neurons. In the corpus callosum, TRIM67 affects axon guidance and tract formation rather than axon branching as observed for TRIM9. Trim67 is also required for growth cone turning in response to Netrin (Boyer et al., 2020).

The opposing functions of Trim9 and Trim67 support a mechanism wherein TRIM67 inhibits the ubiquitination of VASP by Trim9. Through this, and the function of the Netrin-induced deubiquitinase suggested in previous work (Menon et al., 2015), these proteins alter filopodial stability to regulate Netrin-induced attraction (Figure 4A). Of interest, loss of *TRIM67* results in additional defects in adult mice brain. This includes thinning of the hippocampal commissure, as well as decreased brain weight, and decreased area of the hippocampus, the lateral ventricles, and the amygdala. These neurodevelopmental differences may underly decreased learning and altered social novelty behaviors observed in *Trim67* knockout mice (Boyer et al., 2018). In addition to playing a part in axon guidance, these phenotypes may suggest a role for these E3 ligases in additional processes like neuronal migration, proliferation, or survival. This data reveals TRIM9 and TRIM67 as crucial proteins for the fine-tuning of signaling pathways that guide netrin-mediated attraction.

Finally, a more recent study supports a role for Trim9 in regulating axon repulsion in response to Netrin through the Unc-5 receptor. Specifically, high concentrations of Netrin *in vitro* can trigger Unc-5 dependent axon repulsion, and these effects are inhibited in the absence of *trim9* (Mutalik et al., 2025). The precise mechanism through which Trim9 impinges on Unc-5 activity awaits future exploration; however, it is interesting to note that *trim9 and Unc-5C* mutant mice share similar axonal phenotypes in the internal capsule of the brain (Srivatsa et al., 2014; Menon et al., 2015).

#### Slit-mediated repulsion

Slit binding to its receptor Roundabout (Robo) induces repulsion in projecting neurons. In both invertebrates and vertebrates there are three Robo family proteins—Robo1, Robo2, and Robo3— involved in axon guidance (Iversen et al., 2020). While the distinct and overlapping functions of the respective Robo proteins in vertebrates and invertebrates have been reviewed elsewhere, here we will focus exclusively on Robo1 function at the midline (Blockus and Chédotal, 2016). These proteins are well characterized for their function in midline crossing and commissure formation in the invertebrate ventral nerve cord and the vertebrate spinal cord of bilaterally symmetrical organisms. In these structures, Slit is expressed at the midline and the ventral floorplate respectively; however Slit expression coincides with Netrin expression. Therefore, for the crossing commissural neuron (CN) to be selectively permissive to attractive Netrin signaling, CNs must downregulate growth cone expression of Robo1 receptors to prohibit premature Slit-induced repellant signaling. During CN exit of the midline or floorplate, Robo1 surface expression increases, promoting repulsion and preventing re-entry into these regions.

In *Drosophila*, Commissureless (Comm) downregulates Robo1. This occurs through a shunting mechanism in which Comm is expressed in pre-crossing CNs and targets nascent Robo1 for endosomal degradation, preventing its expression at the growth cone membrane (Keleman et al., 2002; Keleman et al., 2005). Loss of Comm leads to a complete loss of commissures and increased Robo1 surface expression (Keleman et al., 2002; Myat et al., 2002). While the requirement of Comm for Robo1 downregulation is accepted, there is conflicting data about how Comm performs this function. One model proposes that Comm downregulates Robo through conserved PY motifs. These motifs would presumably interact with the WW motifs on HECT family E3 ubiquitin ligases, resulting in Comm ubiquitination, and subsequent degradation of the Comm-Robo1 complex. Since expression of Comm variants where these motifs are mutated abolishes Robo1 localization in the late endosome *in vitro* and reduced ectopic midline crossing *in vivo* the importance of the PY motifs is not disputed; however, initial findings determined this to be independent of the HECT E3 ligase Nedd4 (Keleman et al., 2005). In contrast, another report maintains that PY motif-dependent binding of Comm to Nedd4 and Comm ubiquitination are necessary for Robo1 downregulation (Myat et al., 2002).

**FIGURE 5:**
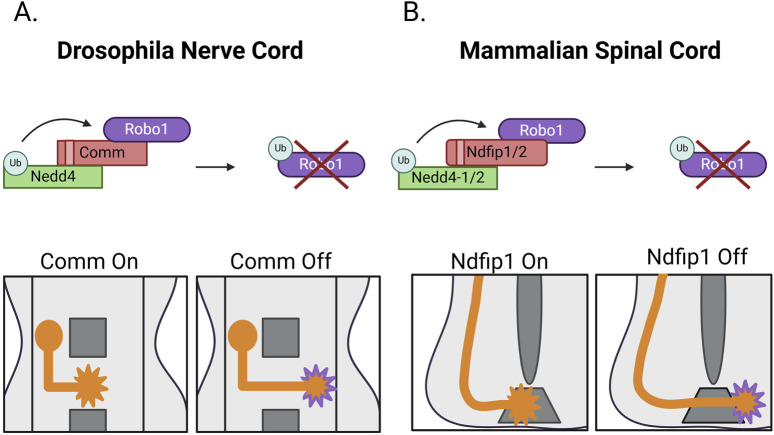
Nedd4-induced Robo1 degradation **(A)** During commissure formation in the *Drosophila* embryonic nerve cord, Comm binds Robo1 and acts as an adaptor to bring Robo1 into proximity with the HECT E3 ligase Nedd4. Through this ternary complex formation, Nedd4 ubiquitinates Robo1, resulting in its endo-lysosomal degradation. Robo1 downregulation prevents nascent Robo1 from reaching the growth cone membrane and impedes premature repulsive signaling in crossing commissural neurons. **(B)** The mammalian spinal cord leverages a similar adaptor-based mechanism during formation of the ventral commissure in which Robo1 binds the adaptors Ndfip1 and/or Ndfip2. These adaptors bind the HECT E3 ligases Nedd4-1 and Nedd4-2. Upon Robo1-Ndfip-Nedd4 complex formation, Robo1 is ubiquitinated and degraded via the endo-lysosomal degradative pathway, preventing Robo1 expression at the growth cone membrane. Post-crossing, Robo1 levels increase at the growth cone to prevent re-entry into the floorplate.

More recently, additional *in vivo* experiments support the requirement of Comm PY motifs for midline crossing. *In vitro* and *in vivo* data demonstrate that Comm PY motifs are required for Robo1 ubiquitination and subsequent downregulation in the lysosome. Comm’s PY motifs are then linked to Comm-mediated Robo1 localization in the late endosome and decreased Robo1 expression at the cell surface both *in vitro* and *in vivo*. Additional data establishes that Comm-dependent Robo1 downregulation is mediated by the formation of a Nedd4/Comm/Robo1 ternary complex. Finally, *in vivo* genetic evidence supports a requirement for Nedd4 in midline crossing. These findings establish a midground between the two previously proposed mechanisms implicating the PY motifs of Comm and Nedd4 in the downregulation of Robo1. In addition to resolving the mechanism of Comm-dependent Robo1 downregulation, this study also puts forth additional information about the role of these PY motifs in the endogenous late endosomal localization of Comm, as Comm colocalization with a late endosomal marker is decreased in PY mutants (Sullivan and Bashaw, 2024) (Figure 5A). It also indicates a PY dose-dependent Comm stabilization, suggesting that the Comm/Nedd4 interaction may be important for Comm downregulation. This is in-line with previous data detailing PY-dependent Comm ubiquitination (Myat et al., 2002). The potential ubiquitination and degradation of Comm by Nedd4 could provide a mechanism to explain the rapid downregulation of Comm in post-crossing axons that triggers increased Robo1 surface expression. This is all the more intriguing given that the mechanism of Comm downregulation remains undefined.

Unlike Slit and its receptor Robo1, Comm is apparently not conserved outside of dipterans, raising the question of how Robo1 receptors are maintained at low levels in pre-crossing commissural axons in the mammalian spinal cord. Interestingly, a similar E3 ubiquitin ligase adaptor-based mechanism for the degradation of mammalian Robo1 receptors was recently discovered (Gorla et al., 2019). Like Comm, Nedd4 Family Interacting Proteins 1 and 2 (Ndfip1/2) are also expressed in commissural neurons of the murine embryonic spinal cord during commissure formation. Ndfip1/2 are known to act as adaptors for HECT family E3 ubiquitin ligases to assist in substrate recruitment via their WW-interacting PY and LPSY motifs. This interaction relieves the autoinhibitory conformation of the E3 ligase, promoting catalytic activity (Mund and Pelham, 2009). Notably, Ndfip proteins interact with Robo1, decrease Robo1 protein levels, and decrease Robo1 surface expression *in vitro*. Expression of Ndfip1/2 also increases Robo1 ubiquitination and degradation in a PY-dependent fashion (Gorla et al., 2019).

*In vivo*, the constitutive knockout of *Ndfip1/2* leads to dose-dependent decreases in commissure thickness at the floor plate in E11.5 mouse embryos. Dye-fill experiments in open-book preparations of the embryonic spinal cord provide more resolution to this reduction in commissure thickness and show that the loss of *Ndfip1/2* leads to increased CN stalling at the floor plate and aberrant ipsilateral turning both pre- and post-crossing. Interestingly, Robo1 protein levels increase in the spinal cord, the brain, and in the ventral commissure of these Ndfip mutant mice during crossing stages. This is in striking contrast to wildtype conditions, where Robo1 protein levels are downregulated until after E12.5 to promote CN crossing. Additionally, Robo1 expression is typically restricted to post-crossing CNs, creating a distinct absence of Robo1 protein at the ventral commissure (Gorla et al., 2019). This elevated expression of Robo1 prior to CN crossing could explain the CN stalling and ventral commissure thinning phenotypes.

After establishing Ndfip1/2 as Comm-like regulators of Robo1 during commissure formation of the mammalian spinal cord, subsequent work connected Ndfip1/2 to an E3 ligase-dependent mechanism of Robo1 lysosomal degradation. As their names indicate, Ndfip1/2 interact with many HECT family E3 ligases, and similarly to Comm, this interaction is dependent on their PY and LPSY motifs. Co-expression of E3 ligases with Ndfip proteins also increases Robo1 ubiquitination and degradation *in vitro*. This effect is dependent on the catalytic activity of E3 ligases as treatment with Heclin, a small molecule inhibitor of the catalytic HECT domain, prevents Nedd4-1/2 mediated Robo1 ubiquitination and degradation. Biochemical data showing that Robo1 ubiquitination is strongly attenuated in mammalian cells expressing both Robo1 and Nedd4 proteins but not Ndfip, reveals that Robo1 ubiquitination relies on the Ndfip1/2-dependent formation of the Robo/Ndfip/Nedd4 ternary complex. In addition, heclin-induced inhibition of HECT E3 ligases in primary CNs increases Slit-induced repulsion, indicating increased Slit responsiveness. *In vivo* Nedd4-1/2 are expressed during stages when CNs are crossing the floor plate, and the loss of *Nedd4-1/*2 in commissural neurons results in thinning of the ventral commissure. The conditional knockdown of *Nedd4-1/*2 also increased CN stalling and failure to reach the floorplate, although to a smaller extent than in *Ndfip1/2* knockout animals (Gorla et al., 2022). The pre-mature repulsion implied by the *in vivo* data, combined with the increased Slit response in heclin-inhibited primary CNs bolsters the model of Ndfip1/2-mediated Robo1 downregulation by Nedd4-1/2 during mammalian commissure formation (Figure 5B). Interestingly, in addition to Ndfip proteins, the PRRG4 protein has also been implicated in the regulation of Robo1 receptors *in vitro*, and in the context of breast cancer tumor metastases, PRRG4 has been shown to regulate Robo1 degradation through recruitment of Nedd4 (Justice et al., 2017; Zhang et al., 2020). Whether PRRG4 or other PRRG proteins regulate Robo1 in the context of axon guidance, in the mammalian spinal cord has not been explored.

Notably, for both Comm and Ndfip1/2, the ability to interact with multiple members of the HECT E3 ligases family does not translate to a role for all binding partners in the regulation of Robo1. In the case of Comm neither Smurf nor Su(dx), the other *Drosophila* HECT E3s, affect commissure formation *in vivo* (Sullivan and Bashaw, 2024). Similarly, only Nedd4-1, Nedd4-2, and WWP1 promote the *in vitro* ubiquitination and degradation of Robo1, despite the fact that other E3 ligases such as Smurf can form a ternary complex with Robo1 and Ndfip proteins (Gorla et al., 2022). These findings suggest there may be an additional layer of regulation between substrate recognition/recruitment and E3 ligase-mediated ubiquitination. These findings reveal the importance of Nedd4 proteins and their adaptors in the regulation of Slit-induced repulsion during midline crossing. Together with their importance in growth-cone attraction, this data identifies E3 ligases as important regulators of axon guidance.

### Section 3: E3 ligases in neurodevelopmental disorders

Neurodevelopmental disorders (NDDs) constitute a diverse group of conditions with NDD patients exhibiting a wide range of neurological and psychological symptoms. According to the most recent edition of the Diagnostic and Statistical Manual of Mental Disorders there are seven categories of NDDs: Autism Spectrum Disorders, Attention-Deficit/Hyperactivity Disorder, Communication Disorders, Intellectual Disorders, Motor Disorders, Specific Learning Disorders, and Tic Disorders (American Psychiatric Association, 2013). These conditions often share common symptoms like cognitive impairment, seizures, mood disorders, social deficits, and varying degrees of motor dysfunction.

The neurodevelopmental field has undertaken the daunting task of attempting to link the genome wide association data derived from patient samples back to basic science to gain insight into the pathological mechanisms behind these disorders. Over time, one of the common themes that has emerged from this research is the important role of E3 ubiquitin ligases and the disruption of ubiquitin-induced protein degradation in the pathogenesis of NDDs (Wang Y. et al., 2020; Mabb, 2021; Krzeski et al., 2024). In this section, we will connect our discussion of the broader neurodevelopmental functions of E3 ligases like neuronal differentiation, axon guidance, and dendrite morphogenesis, with recent discoveries that shed light on the neurodevelopmental root of some NDDs (Table 1). This discussion is not intended to be exhaustive and only serves to highlight a few RING and HECTE3 ligases with well-defined mechanisms of specific substrate regulation in the context of NDDs.

**TABLE 1:** Summary of discussed E3 ligases, substrates, and functions in Neurodevelopment.

Neurodevelopmental Process	E3 Ligase	Substrate	Neurodevelopmental Role	Associated NDD	References
Neural Differentiation	RNF220	Gli2/3	Shh gene transcription in spinal cord and hindbrain		Ma et al. (2019)
	CUL4A	Sox2	Neural progenitor gene transcription		Cui et al. (2018)
	WWP2	Sox2	Neural progenitor gene transcription		Fang et al. (2014)
	HUWE1	p53	Neural progenitor gene transcription	Juberg-Marsidi Syndrome	Aprigliano et al. (2021)
	RNF12/Rlim	Rex1	Embryonic stem cell gene transcription	X-linked Intellectual Disability	Bustos et al. (2018)
Axon Guidance	TRIM9	VASP	Netrin-mediated filopodia extension		Menon et al. (2015)
	TRIM9	Dcc	Netrin-mediated FAK signaling	^a^Congenital Mirror Movement Disorder	Plooster et al. (2017)
	TRIM67	Trim9	Netrin-mediated filopodia extension		Boyer et al. (2020)
	NEDD4	Robo1	Slit-mediated repulsion during midline crossing	^a^Horizontal Gaze Palsy	Gorla et al. (2022), Sullivan and Bashaw (2024)
Dendritic Morphology	CRL4	Dcx	Dendrite & axon outgrowth	X-linked Intellectual Disability	Shim et al. (2024)
	TRIM32	CDYL	Dendrite arborization; BDNF signaling	Autism Spectrum Disorder	Liu et al. (2022)
	UBE3A	HAP1	Autophagy during dendritic spine formation	Angelman Syndrome	Wang et al. (2019a)
	UBE3A	XIAP	Caspase3-mediated dendritic pruning	Autism Spectrum Disorder	Khatri et al. (2018)

^a^ The direct involvement of E3 ligase regulation in the pathogenesis of this NDD is unclear

#### Angelman Syndrome

Angelman Syndrome (AS) is a neuro-genetic disorder affecting 1 in 15,000 individuals that becomes apparent within the first year of life. Symptoms of AS include developmental delay, recurring seizures, movement disorders, sleep problems, and severe speech impairment. AS patient studies have revealed some of the underlying molecular mechanisms for the pathogenesis of AS that implicate mutations in the gene encoding UBE3A, a HECT E3 ligase. Some loss of function mutations decrease UBE3A expression and result in impaired dendritic spine development, while other variants are instead reported to decrease the E3 ligase activity of UBE3A (Kishino et al., 1997; Cooper et al., 2004; Dindot et al., 2007; Margolis et al., 2015; Beasley et al., 2020).

Interestingly, *in vitro* data supports an interaction between UBE3A and Huntingtin-associated protein 1 (HAP1), a protein expressed in the brain that has primarily been studied in the context of neurodegenerative disorders. In neurodegeneration, HAP1 is implicated in retrograde autophagosome transport and subsequent fusion with competent lysosomes (Maday et al., 2012; Wong and Holzbaur, 2014). Selective autophagy is a homeostatic process in which the autophagosome degrades organelles and other protein cargoes through fusion with the lysosome. In mice modelling the neurodevelopmental loss of function caused by *UBE3A* patient mutations, there is an increase in HAP1 protein expression, a decrease in HAP1 ubiquitination, and an increase in autophagy (Wang T. et al., 2019). *In vitro* assays in cells derived from *UBE3A* mutants and in cell lines expressing inactive forms of UBE3A affirmed that similar increases in autophagy were due to decreased HAP1 ubiquitination and its subsequent over-expression. The aberrant dendritic spine morphology seen in AS models, may also be linked to increased autophagy since pharmacological inhibition of autophagy rescues morphological and some behavioral phenotypes associated with these models; however, there is currently no direct connection between the HAP1 over-expression observed in AS neurons, and AS pathology (Wang T. et al., 2019).

By determining how HAP1 increases autophagy it may be possible to establish a causal link between AS and UBE3A loss of function. In the early stages of autophagy, autophagic receptors (ARs) bind membrane-bound autophagy-related (ATG) proteins that are important for the formation of the phagophore and the initiation of autophagy. Specifically, ATG14 is important for the formation of the PtdIns3-kinase (PtdIns3K) complex, and upon binding, targets the complex to the pre-autophagosome (Obara and Ohsumi, 2011). This targeting results in the formation of PtdIns3P, a lipid essential for the recruitment of additional autophagy machinery to the autophagosome (Brier et al., 2019). ARs also bind ubiquitinated cargo to mediate their incorporation into the autophagosome. (Münch and Dikic, 2018; Liénard et al., 2024). Data reporting HAP1-ATG14 associations also connects UBE3A loss of function with recruitment of the PtdIns3K complex, which enhances PtdIns3P formation, and increases autophagosome assembly (Wang T. et al., 2019; Nishimura and Tooze, 2020). These findings both expand the role of HAP1 in autophagy to neurodevelopment and provide insights into AS pathology. They also implicate HAP1 in autophagosome assembly, rather than its function in autophagosome transport and motor association that are linked to neurodegeneration. Additionally, PtdIns3P on the autophagosome recruits Tectonin domain-containing protein 1 (TECPR1), a protein required to induce autophagosome-lysosome fusion (Chen et al., 2012; Terawaki et al., 2015). HAP1 facilitating PtdIns3P formation and potentially recruitment of TECPR1 could also place HAP1 upstream of an autophagosome-lysosome fusion pathway. Given the recent discovery of various neural UBE3A substrates (Krzeski et al., 2024), these insights highlight just one example of UBE3A as a key factor in the dysregulation of autophagy that is associated with the pathophysiology of AS.

#### Autism spectrum disorders

Autism Spectrum Disorders (ASD) are highly heritable, polygenetic disorders that are frequently characterized by social and language impairments and repetitive behaviors. According to the CDC, 1 in 36 children was diagnosed with ASD in 2020, with males being four times more likely to be diagnosed than females (Maenner et al., 2023).

UBE3A (also known as E6AP) is also linked to ASD susceptibility. While loss of function mutations in *UBE3A* are linked to AS symptoms, duplications and triplications of *UBE3A* are associated with ASD. The expression of only the maternal copy of *UBE3A* in the cerebral cortex and in Purkinje neurons in the cerebellum reinforces the importance of *UBE3A* dosage control in the brain (Albrecht et al., 1997; Hogart et al., 2010; Roy et al., 2023). In addition to increases in UBE3A copy number, a *de novo* autism-linked missense variant that leads to elevated UBE3A activity has also been identified (Yi et al., 2015). This specific mutation renders UBE3A resistant to normal inhibition by protein kinase A (PKA) phosphorylation, resulting in excessive E3 ligase activity. PKA inhibition of UBE3A appears to underly the effect of PKA on cortical neuron dendrite morphogenesis, since the increases in dendritic spine density observed upon chronic inhibition of PKA in primary cortical neurons is lost in UBE3A mutant neurons. This indicates that PKA’s negative regulation of UBE3A may normally act to constrain dendritic formation. Interestingly, mis-expression of this “active” variant of UBE3A by *in utero* electroporation leads to a significant increase in dendritic spine density in layer 2/3 pyramidal neurons *in vivo* (Yi et al., 2015); however, the UBE3A substrates that account for the increased spine density remain to be explored.

In direct contrast to these findings, a more recent study reported that over-expression of UBE3A in primary neurons and elevated UBE3A expression in an ASD mouse model that carries three copies of the normal UBE3A gene leads to the opposite effect, a decrease in dendritic spine length and complexity. The effects of UBE3A over-expression coincide with increased levels of active caspase-3 (Khatri et al., 2018), which has previously been shown to promote dendritic pruning. UBE3A leads to the elevation of active caspase-3 by targeting its upstream inhibitor X-linked inhibitor of apoptosis protein (XIAP) for ubiquitination and degradation (Scott et al., 2005; D’Amelio et al., 2010). Consistent with this idea, expression of XIAP rescues the reduction in dendritic spine length and complexity in primary neurons over-expressing UBE3A (Khatri et al., 2018). Curiously, the UBE3A-dependent decrease in dendritic complexity is consistent with the earlier observation that UBE3A over-expression in hippocampal slice culture leads to reduction in synaptic transmission; however, in this study no effects on dendrite morphology were reported (Smith et al., 2011).

While the explanation for these discordant findings on the effects of UBE3A over-expression on cortical dendrite morphogenesis and spine density is unclear, there are many differences in the ways these studies were performed that make direct comparisons difficult. For example, two of these groups used UBE3A mice that carry triplication of the locus to achieve over-expression (Smith et al., 2011; Khatri et al., 2018), while the other used *in utero* electroporation (Yi et al., 2015); thus, the timing and levels of over-expression varied between the studies. In addition, the specific neurons examined differed in layer location and level of maturity, and there were differences in the ways dendritic structures were categorized. Regardless of these apparent discrepancies on the role of UBE3A, these observations indicate that the association of elevated UBE3A with ASD is correlated with changes in dendritic complexity and spine density and/or synaptic function. In addition, key UBE3A substrates that may contribute to these effects have begun to be identified, forming the foundation for future investigation.

In addition to UBE3A, mutations in RING E3 ligase Tripartate motif-containing protein 32 (TRIM32) increase risk for ASD and knockout of TRIM32 in mouse models results in an ASD-like phenotype (Zhu et al., 2021). Recent data proposes a role for TRIM32 in the regulation of Chromodomain Y-like (CDYL), a chromatin-binding protein that recruits histone methyltransferases to inhibit downstream gene transcription (Zhang et al., 2011; Wang M. et al., 2020). Specifically, CDYL interaction with Polycomb Repressive Complex (PRC2) and the subsequent recruitment of H3K27 methyltransferase to the promoter of brain-derived neurotrophic factor (BDNF) inhibits BDNF (Qi et al., 2014). This decreases BDNF binding to TrkB receptor tyrosine kinase and attenuates MAPK signaling important for dendritic growth (Finsterwald et al., 2010).

Biochemical data using proteins purified from rat brains demonstrates that TRIM32 interacts with CDYL through its N and C-termini. *In vitro* data reports that this results in CDYL ubiquitination and proteasomal degradation. TRIM32 over-expression in cultured hippocampal neurons significantly increases dendritic branching in a catalytic domain-dependent fashion, while shRNA-induced knockdown of TRIM32 decreases dendritic branching. This affect is CDYL-dependent, placing TRIM32 upstream of CDYL-mediated dendritic arborization (Liu et al., 2022). Further investigation of the impact of TRIM32 manipulation on BDNF transcription would cement this connection. The high density of dendritic spines in Purkinje neurons, combined with the developmental expression of TRIM32 and CDYL in the cerebellum may imply a generalized function for TRIM32 in dendritic arborization (Wang M. et al., 2020). TRIM32 seems to impact the formation of dendritic spines in the adult brain as well, marking a potential for sustained TRIM32 function (Zhu et al., 2021). These findings indicate an indispensable role for CRL4 and TRIM32 in orchestrating dendritic outgrowth.

#### X-linked intellectual disability

X-linked intellectual disability (XLID) is a broad term for over 150 different syndromes and more non-syndromic forms. Over 100 genetic mutations account for the syndromic forms alone, making them highly heterogeneous disorders (Lubs et al., 2012; Stevenson et al., 2012). Due to this marked heterogeneity, the clinical features of XLID vary, but they are commonly defined by impairment of mental abilities that alter adaptive conceptual, social, or practical skills (American Psychiatric Association, 2013). XLID is thought to arise from abnormalities in neural differentiation, neurite projection and dendritic spine formation due to the cortical differences observed in patients with XLID (Bassani et al., 2013; Telias and Ben-Yosef, 2014).

RNF12/Rlim is a RING E3 ligase associated with XLID. RNF12/Flim regulates neural gene expression through REX1 degradation and X-chromosome inactivation (Jonkers et al., 2009; Bustos et al., 2018; Frints et al., 2019; Wang and Bach, 2019). XLID-associated mutations in RNF12/Rlim are found in the basic region and the RING domain of the protein. *In vitro* experiments in cultured ESCs expressing the XLID RNF12/Rlim mutations results in decreased ubiquitination of its known substrates, REX1 and Smad7, due to decreased catalytic activity. Based on data recapitulating this decreased catalytic activity, accelerations in neural differentiation, and abnormal ESC differentiation in a knock-in mouse model, alterations in RNF12/Rlim-mediated ubiquitination could be the mechanism of pathology caused by these mutations in XLID patients (Bustos et al., 2018).

The HECT E3 ligase HUWE1 is also genetically linked to XLID and plays an important role in the neuronal and glial differentiation of NPCs in mice (Zhao et al., 2008; Friez et al., 2016; Giles and Grill, 2020; Muthusamy et al., 2020). Since HUWE1 regulates p53 in non-neuronal cells, and p53 is also linked to the NSC metabolic balance and neuronal differentiation, it is postulated that a similar mechanism could be at play in neurodevelopment (Yang et al., 2018; Marin Navarro et al., 2020). Interestingly *de novo* mutations in human patients with XLID have been traced to point mutations in the HECT domain and other regions of HUWE1. These mutations result in the upregulation of members in the p53 signaling pathway. A severe form of XLID called Juberg-Marsidi Syndrome (JMS), is characterized by a G4310R point mutation within the HUWE1 HECT domain (Friez et al., 2016). Despite the location of the mutation implying a possible difference in catalytic activity, the mutation seems to instead alter protein stability, resulting in decreased expression. In this context, it is interesting to note that previous work on several other HECT family proteins including Itch, WWP1, and WWP2 indicates that HECT-WW domain interactions can confer autoinhibition (Wang Z. et al., 2019). When this intramolecular binding is perturbed, these HECT ligases display increased autoubiquitination and decreases in protein stability (Wang Z. et al., 2019). It remains to be explored whether the G4310R JMS mutant in HUWE1 upregulates the p53 pathway by reducing the binding affinity between HUWE1 and p53, or alternatively by leading to the autoubiquitination and degradation of HUWE1 itself.

Induced pluripotent stem cells cultured from patients with the G4310R point mutation, display an accumulation and excessive activation of p53, increased expression of CDKN1A/p21, and a concordant decrease in neural differentiation. Using patient-derived HUWE1 mutations, these findings support a causal link between the pathological neural differentiation impairment of JMS and aberrant regulation of the p53 signaling pathway caused by decreased HUWE1 stability (Aprigliano et al., 2021). These discoveries reveal functions for RNF12/Rlim and HUWE1 in the atypical neural differentiation found in XLID, and JMS respectively.

Mutations in the CUL4B loci are also linked to XLID (Zou et al., 2007). As previously discussed, Cullin Ring Ligase 4 complex (CRL4) can refer to a Cul4a-containing E3 ligase complex; however, CRL4 can also form with a Cul4b core, creating a similar but distinct complex. Interestingly, gene ontology and interactome analysis on cultured rat cortical neurons show interaction of Cul4a/b with several cytoskeletal proteins, including Doublecortin (Dcx), a microtubule associated protein (MAP) (Shim et al., 2024). Dcx stabilizes microtubules, facilitating their polymerization for the formation of exploratory axonal and dendritic extensions that will eventually synapse with surrounding neurons and form functional circuits (Parato and Bartolini, 2021). The potential importance of this protein’s regulation in neurodevelopment are underpinned by the causative link of *Dcx* mutations in X-linked lissencephaly (Fu et al., 2013).

In addition to interaction, CRL4 ubiquitinates and downregulates Dcx *in vitro. In vitro* knockout of Cul4a and Cul4b resulted in longer, more complex neurites and dendrites, presumably through increased microtubule stability from sustained Dcx expression and activity. In cortical neuron cultures, activation of Cul4a/b is initiated by neddylation, and occurs early in neurodevelopment. Over-expression of Cul4a/b variants that cannot bind to their RING finger subunit or be activated by neddylation only increased neurite outgrowth in the Cul4a condition and increased dendritic branching in both conditions. Furthermore, *in vitro* over-expression of Cul4a alone decreases axonal and dendritic outgrowth, while Cul4b over-expression has no effect. This supports a mechanism in which CRL4a and CRL4b regulation of Dcx may differentially regulate axonal and dendritic outgrowth (Shim et al., 2024).

DCX is also ubiquitinated and degraded by Kelch-like 15 (KLHL15), a substrate-adaptor of the CRL3 complex. *In vitro* data indicate that DCX-KLHL15 ubiquitination depends on the DCX FRY domain. Like CRL4, the expression of KLHL15 antagonizes dendritic outgrowth in the presence of DCX (Song et al., 2021). Despite the previously identified mutations in DCX that are associated with X-linked intellectual disability seeming to be outside of its FRY domain, the similarity of key players and phenotypes might suggest that further investigation of potential link between key regulators of DCX and X-linked intellectual disability (Matsumoto et al., 2001).

### Section 4: Future directions

Over the last several years, research has expanded our understanding of E3 ligases, implicating them in diverse neurodevelopmental processes. Advances in genetic tools and access to patient genomic data have also revealed roles for E3 ligases in the etiology of neurodevelopmental disorders. Nevertheless, many questions remain. In the case of Nedd4 and the regulation of the Robo1 receptor, Ndfip-dependent recruitment of HECT ligases to the receptor is necessary but not sufficient to trigger Robo1 ubiquitination. Specifically, both Smurf and Nedd4 can form a ternary complex with Robo1 and Ndfip proteins *in vitro*, but only Nedd4 can drive Robo1 degradation. This raises the important question of what distinguishes the substrate specificity of an E3 ligase from its functional specificity? Better understanding of this area may also provide structural information, enabling modulation of E3 ligase substrate interaction and E3 ligase function. Another important area for future investigation is the mechanism underlying differential E3 ligase expression and activation that can confer cell-type or temporal control of target protein activities, as exemplified by differential Sox2 regulation in NPCs versus ESCs.

Moreover, many of the E3 ligases discussed here have multiple functions throughout neurodevelopment; however, it remains to be seen if these proteins have important neuronal functions throughout life or in processes like neurodegeneration. This could inform whether NDD phenotypes in adults, like decreased synapse number in adults with ASD, primarily arise from developmental deficits, or if E3 ligase mutations continue to cause aberrations into adulthood, due to sustained requirements for these proteins in neuronal homeostasis (Matuskey et al., 2024). Continuing to leverage genomic data to direct the mechanistic analysis of NDD-associated E3 ligase mutations is also an important area for future work. Approaching the mechanism from the perspective of known NDD-associated proteins or pathways and determining their ability to interact with additional E3 ligases could also yield new insights.

Lastly, with so many E3 ligases encoded in the human genome, and many of them having more than one name, it will be beneficial to construct a consolidated interactive repository of E3 ligase substrates and spatiotemporal expression patterns in the central nervous system. Currently, expression and substrate data are divided between databases like ELiAH and UbiNet2.0, with a notable absence of temporal expression and all CNS expression data on ELiAH (Li et al., 2021; Paik et al., 2024). In all, continued exploration of novel E3 ligase substrates will improve understanding of how substrate ubiquitination leads to protein degradation, guides localization, and regulates alternative post-translational modification. This understanding will undoubtedly elucidate mechanisms important for neurodevelopment and the molecular basis of NDDs, but also in biological contexts outside of the nervous system.

## References

[B1] Ahel J. Fletcher A. Grabarczyk D. B. Roitinger E. Deszcz L. Lehner A. (2021). E3 ubiquitin ligase RNF213 employs a non-canonical zinc finger active site and is allosterically regulated by ATP. 10.1101/2021.05.10.443411

[B2] Akutsu M. Dikic I. Bremm A. (2016). Ubiquitin chain diversity at a glance. J. Cell Sci. 129, 875–880. 10.1242/jcs.183954 26906419

[B3] Alberts B. Johnson A. Lewis J. Raff M. Roberts K. Walter P. (2002). in Molecular biology of the cell (New York: Garland Science).

[B4] Albrecht U. Sutcliffe J. S. Cattanach B. M. Beechey C. V. Armstrong D. Eichele G. (1997). Imprinted expression of the murine Angelman syndrome gene, Ube3a, in hippocampal and Purkinje neurons. Nat. Genet. 17, 75–78. 10.1038/ng0997-75 9288101

[B5] American Psychiatric Association (2013). Diagnostic and statistical manual of mental disorders. Fifth Edition. American Psychiatric Association. 10.1176/appi.books.9780890425596

[B6] Aprigliano R. Aksu M. E. Bradamante S. Mihaljevic B. Wang W. Rian K. (2021). Increased p53 signaling impairs neural differentiation in HUWE1-promoted intellectual disabilities. Cell Rep. Med. 2, 100240. 10.1016/j.xcrm.2021.100240 33948573 PMC8080178

[B7] Bassani S. Zapata J. Gerosa L. Moretto E. Murru L. Passafaro M. (2013). The neurobiology of X-linked intellectual disability. Neuroscientist 19, 541–552. 10.1177/1073858413493972 23820068

[B8] Beasley S. A. Kellum C. E. Orlomoski R. J. Idrizi F. Spratt D. E. (2020). An Angelman syndrome substitution in the HECT E3 ubiquitin ligase C-terminal Lobe of E6AP affects protein stability and activity. PLoS ONE 15, e0235925. 10.1371/journal.pone.0235925 32639967 PMC7343168

[B9] Blockus H. Chédotal A. (2016). Slit-Robo signaling. Development 143, 3037–3044. 10.1242/dev.132829 27578174

[B10] Boyer N. P. McCormick L. E. Menon S. Urbina F. L. Gupton S. L. (2020). A pair of E3 ubiquitin ligases compete to regulate filopodial dynamics and axon guidance. J. Cell Biol. 219, e201902088. 10.1083/jcb.201902088 31820781 PMC7039193

[B11] Boyer N. P. Monkiewicz C. Menon S. Moy S. S. Gupton S. L. (2018). Mammalian TRIM67 functions in brain development and behavior. eNeuro 5, 0186–218. 10.1523/ENEURO.0186-18.2018 PMC6002264 29911180

[B12] Brier L. W. Ge L. Stjepanovic G. Thelen A. M. Hurley J. H. Schekman R. (2019). Regulation of LC3 lipidation by the autophagy-specific class III phosphatidylinositol-3 kinase complex. Mol. Biol. Cell 30, 1098–1107. 10.1091/mbc.E18-11-0743 30811270 PMC6724508

[B13] Buckley S. M. Aranda-Orgilles B. Strikoudis A. Apostolou E. Loizou E. Moran-Crusio K. (2012). Regulation of pluripotency and cellular reprogramming by the ubiquitin-proteasome system. Cell Stem Cell 11, 783–798. 10.1016/j.stem.2012.09.011 23103054 PMC3549668

[B14] Bustos F. Segarra-Fas A. Chaugule V. K. Brandenburg L. Branigan E. Toth R. (2018). RNF12 X-linked intellectual disability mutations disrupt E3 ligase activity and neural differentiation. Cell Rep. 23, 1599–1611. 10.1016/j.celrep.2018.04.022 29742418 PMC5976579

[B15] Chaudhari K. Zhang K. Yam P. T. Zang Y. Kramer D. A. Gagnon S. (2024). A human DCC variant causing mirror movement disorder reveals that the WAVE regulatory complex mediates axon guidance by netrin-1–DCC. Sci. Signal. 17, eadk2345. 10.1126/scisignal.adk2345 39353037 PMC11568466

[B16] Chédotal A. (2019). Roles of axon guidance molecules in neuronal wiring in the developing spinal cord. Nat. Rev. Neurosci. 20, 380–396. 10.1038/s41583-019-0168-7 31000796

[B17] Chen D. Fan W. Lu Y. Ding X. Chen S. Zhong Q. (2012). A mammalian autophagosome maturation mechanism mediated by TECPR1 and the atg12-atg5 conjugate. Mol. Cell 45, 629–641. 10.1016/j.molcel.2011.12.036 22342342 PMC3299828

[B18] Cheng J. Bin X. Tang Z. (2024). Cullin-RING ligase 4 in cancer: structure, functions, and mechanisms. Biochimica Biophysica Acta (BBA) - Rev. Cancer 1879, 189169. 10.1016/j.bbcan.2024.189169 39117093

[B19] Clague M. J. Heride C. Urbé S. (2015). The demographics of the ubiquitin system. Trends Cell Biol. 25, 417–426. 10.1016/j.tcb.2015.03.002 25906909

[B20] Cooper E. M. Hudson A. W. Amos J. Wagstaff J. Howley P. M. (2004). Biochemical analysis of angelman syndrome-associated mutations in the E3 ubiquitin ligase E6-associated protein. J. Biol. Chem. 279, 41208–41217. 10.1074/jbc.M401302200 15263005

[B21] Cowan A. D. Ciulli A. (2022). Driving E3 ligase substrate specificity for targeted protein degradation: lessons from nature and the laboratory. Annu. Rev. Biochem. 91, 295–319. 10.1146/annurev-biochem-032620-104421 35320687

[B22] Cui C.-P. Zhang Y. Wang C. Yuan F. Li H. Yao Y. (2018). Dynamic ubiquitylation of Sox2 regulates proteostasis and governs neural progenitor cell differentiation. Nat. Commun. 9, 4648. 10.1038/s41467-018-07025-z 30405104 PMC6220269

[B23] D’Amelio M. Cavallucci V. Cecconi F. (2010). Neuronal caspase-3 signaling: not only cell death. Cell Death Differ. 17, 1104–1114. 10.1038/cdd.2009.180 19960023

[B24] Dessaud E. McMahon A. P. Briscoe J. (2008). Pattern formation in the vertebrate neural tube: a sonic hedgehog morphogen-regulated transcriptional network. Development 135, 2489–2503. 10.1242/dev.009324 18621990

[B25] Dikic I. Wakatsuki S. Walters K. J. (2009). Ubiquitin-binding domains - from structures to functions. Nat. Rev. Mol. Cell Biol. 10, 659–671. 10.1038/nrm2767 19773779 PMC7359374

[B26] Dindot S. V. Antalffy B. A. Bhattacharjee M. B. Beaudet A. L. (2007). The Angelman syndrome ubiquitin ligase localizes to the synapse and nucleus, and maternal deficiency results in abnormal dendritic spine morphology. Hum. Mol. Genet. 17, 111–118. 10.1093/hmg/ddm288 17940072

[B27] Drees F. Gertler F. B. (2008). Ena/VASP: proteins at the tip of the nervous system. Curr. Opin. Neurobiol. 18, 53–59. 10.1016/j.conb.2008.05.007 18508258 PMC2515615

[B28] Evans T. A. Bashaw G. J. (2010). Axon guidance at the midline: of mice and flies. Curr. Opin. Neurobiol. 20, 79–85. 10.1016/j.conb.2009.12.006 20074930 PMC4128228

[B29] Fang L. Zhang L. Wei W. Jin X. Wang P. Tong Y. (2014). A methylation-phosphorylation switch determines Sox2 stability and function in ESC maintenance or differentiation. Mol. Cell 55, 537–551. 10.1016/j.molcel.2014.06.018 25042802

[B30] Favaro R. Valotta M. Ferri A. L. M. Latorre E. Mariani J. Giachino C. (2009). Hippocampal development and neural stem cell maintenance require Sox2-dependent regulation of Shh. Nat. Neurosci. 12, 1248–1256. 10.1038/nn.2397 19734891

[B31] Finci L. I. Krüger N. Sun X. Zhang J. Chegkazi M. Wu Y. (2014). The crystal structure of netrin-1 in complex with DCC reveals the bifunctionality of netrin-1 as a guidance cue. Neuron 83, 839–849. 10.1016/j.neuron.2014.07.010 25123307 PMC4412161

[B32] Finsterwald C. Fiumelli H. Cardinaux J.-R. Martin J.-L. (2010). Regulation of dendritic development by BDNF requires activation of CRTC1 by glutamate. J. Biol. Chem. 285, 28587–28595. 10.1074/jbc.M110.125740 20639200 PMC2937884

[B33] Friez M. J. Brooks S. S. Stevenson R. E. Field M. Basehore M. J. Adès L. C. (2016). HUWE1 mutations in Juberg-Marsidi and Brooks syndromes: the results of an X-chromosome exome sequencing study. BMJ Open 6, e009537. 10.1136/bmjopen-2015-009537 PMC4854010 27130160

[B34] Frints S. G. M. Ozanturk A. Rodríguez Criado G. Grasshoff U. de Hoon B. Field M. (2019). Pathogenic variants in E3 ubiquitin ligase RLIM/RNF12 lead to a syndromic X-linked intellectual disability and behavior disorder. Mol. Psychiatry 24, 1748–1768. 10.1038/s41380-018-0065-x 29728705

[B35] Fu X. Brown K. J. Yap C. C. Winckler B. Jaiswal J. K. Liu J. S. (2013). Doublecortin (Dcx) family proteins regulate filamentous actin structure in developing neurons. J. Neurosci. 33, 709–721. 10.1523/JNEUROSCI.4603-12.2013 23303949 PMC3711551

[B36] Gaspard N. Vanderhaeghen P. (2010). Mechanisms of neural specification from embryonic stem cells. Curr. Opin. Neurobiol. 20, 37–43. 10.1016/j.conb.2009.12.001 20080043

[B37] Giles A. C. Grill B. (2020). Roles of the HUWE1 ubiquitin ligase in nervous system development, function and disease. Neural Dev. 15, 6. 10.1186/s13064-020-00143-9 32336296 PMC7184716

[B38] Gorla M. Chaudhari K. Hale M. Potter C. Bashaw G. J. (2022). A Nedd4 E3 Ubiquitin ligase pathway inhibits Robo1 repulsion and promotes commissural axon guidance across the midline. J. Neurosci. JN-RM, 7547–7561. 10.1523/JNEUROSCI.2491-21.2022 PMC9546450 36002265

[B39] Gorla M. Santiago C. Chaudhari K. Layman A. A. K. Oliver P. M. Bashaw G. J. (2019). Ndfip proteins target Robo receptors for degradation and allow commissural axons to cross the midline in the developing spinal cord. Cell Rep. 26, 3298–3312.e4. 10.1016/j.celrep.2019.02.080 30893602 PMC6913780

[B40] Graham V. Khudyakov J. Ellis P. Pevny L. (2003). SOX2 functions to maintain neural progenitor identity. Neuron 39, 749–765. 10.1016/s0896-6273(03)00497-5 12948443

[B41] Haas A. L. Warms J. V. Hershko A. Rose I. A. (1982). Ubiquitin-activating enzyme. Mechanism and role in protein-ubiquitin conjugation. J. Biol. Chem. 257, 2543–2548. 10.1016/s0021-9258(18)34958-5 6277905

[B42] Harris R. Sabatelli L. M. Seeger M. A. (1996). Guidance cues at the Drosophila CNS midline: identification and characterization of two Drosophila netrin/UNC-6 homologs. Neuron 17, 217–228. 10.1016/S0896-6273(00)80154-3 8780646

[B43] Hershko A. Ciechanover A. (1998). The ubiquitin system. Annu. Rev. Biochem. 67, 425–479. 10.1146/annurev.biochem.67.1.425 9759494

[B44] Hogart A. Wu D. LaSalle J. M. Schanen N. C. (2010). The comorbidity of autism with the genomic disorders of chromosome 15q11.2-q13. Neurobiol. Dis. 38, 181–191. 10.1016/j.nbd.2008.08.011 18840528 PMC2884398

[B45] Horn-Ghetko D. Krist D. T. Prabu J. R. Baek K. Mulder M. P. C. Klügel M. (2021). Ubiquitin ligation to F-box protein targets by SCF–RBR E3–E3 super-assembly. Nature 590, 671–676. 10.1038/s41586-021-03197-9 33536622 PMC7904520

[B46] Hui C. C. Slusarski D. Platt K. A. Holmgren R. Joyner A. L. (1994). Expression of three mouse homologs of the Drosophila segment polarity gene cubitus interruptus, Gli, Gli-2, and Gli-3, in ectoderm- and mesoderm-derived tissues suggests multiple roles during postimplantation development. Dev. Biol. 162, 402–413. 10.1006/dbio.1994.1097 8150204

[B47] Iversen K. Beaubien F. Prince J. E. A. Cloutier J.-F. (2020). “Axon guidance: slit–robo signaling,” in Cellular migration and formation of axons and dendrites (Elsevier), 147–173. 10.1016/B978-0-12-814407-7.00007-9

[B48] Jevtić P. Haakonsen D. L. Rapé M. (2021). An E3 ligase guide to the galaxy of small-molecule-induced protein degradation. Cell Chem. Biol. 28, 1000–1013. 10.1016/j.chembiol.2021.04.002 33891901

[B49] Johnson E. S. Ma P. C. Ota I. M. Varshavsky A. (1995). A proteolytic pathway that recognizes ubiquitin as a degradation signal. J. Biol. Chem. 270, 17442–17456. 10.1074/jbc.270.29.17442 7615550

[B50] Jonkers I. Barakat T. S. Achame E. M. Monkhorst K. Kenter A. Rentmeester E. (2009). RNF12 is an X-encoded dose-dependent activator of X chromosome inactivation. Cell 139, 999–1011. 10.1016/j.cell.2009.10.034 19945382

[B51] Justice E. D. Barnum S. J. Kidd T. (2017). The WAGR syndrome gene PRRG4 is a functional homologue of the commissureless axon guidance gene. PLoS Genet. 13, e1006865. 10.1371/journal.pgen.1006865 28859078 PMC5578492

[B52] Kawabe H. Stegmüller J. (2021). The role of E3 ubiquitin ligases in synapse function in the healthy and diseased brain. Mol. Cell. Neurosci. 112, 103602. 10.1016/j.mcn.2021.103602 33581237

[B53] Keleman K. Rajagopalan S. Cleppien D. Teis D. Paiha K. Huber L. A. (2002). Comm sorts Robo to control axon guidance at the Drosophila midline. Cell 110, 415–427. 10.1016/S0092-8674(02)00901-7 12202032

[B54] Keleman K. Ribeiro C. Dickson B. J. (2005). Comm function in commissural axon guidance: cell-autonomous sorting of Robo *in vivo* . Nat. Neurosci. 8, 156–163. 10.1038/nn1388 15657595

[B55] Kerscher O. Felberbaum R. Hochstrasser M. (2006). Modification of proteins by ubiquitin and ubiquitin-like proteins. Annu. Rev. Cell Dev. Biol. 22, 159–180. 10.1146/annurev.cellbio.22.010605.093503 16753028

[B56] Khatri N. Gilbert J. P. Huo Y. Sharaflari R. Nee M. Qiao H. (2018). The autism protein Ube3A/e6ap remodels neuronal dendritic arborization via caspase-dependent microtubule destabilization. J. Neurosci. 38, 363–378. 10.1523/JNEUROSCI.1511-17.2017 29175955 PMC5761614

[B57] Kim H. C. Steffen A. M. Oldham M. L. Chen J. Huibregtse J. M. (2011). Structure and function of a HECT domain ubiquitin-binding site. EMBO Rep. 12, 334–341. 10.1038/embor.2011.23 21399621 PMC3077248

[B58] Kishino T. Lalande M. Wagstaff J. (1997). UBE3A/E6-AP mutations cause Angelman syndrome. Nat. Genet. 15, 70–73. 10.1038/ng0197-70 8988171

[B59] Komander D. Rape M. (2012). The ubiquitin code. Annu. Rev. Biochem. 81, 203–229. 10.1146/annurev-biochem-060310-170328 22524316

[B60] Krzeski J. C. Judson M. C. Philpot B. D. (2024). Neuronal UBE3A substrates hold therapeutic potential for Angelman syndrome. Curr. Opin. Neurobiol. 88, 102899. 10.1016/j.conb.2024.102899 39126903 PMC11397222

[B61] Lebrand C. Dent E. W. Strasser G. A. Lanier L. M. Krause M. Svitkina T. M. (2004). Critical role of Ena/VASP proteins for filopodia formation in neurons and in function downstream of netrin-1. Neuron 42, 37–49. 10.1016/s0896-6273(04)00108-4 15066263

[B62] Li W. Lee J. Vikis H. G. Lee S.-H. Liu G. Aurandt J. (2004). Activation of FAK and Src are receptor-proximal events required for netrin signaling. Nat. Neurosci. 7, 1213–1221. 10.1038/nn1329 15494734 PMC2373267

[B63] Li Z. Chen S. Jhong J.-H. Pang Y. Huang K.-Y. Li S. (2021). UbiNet 2.0: a verified, classified, annotated and updated database of E3 ubiquitin ligase–substrate interactions. Database 2021, baab010. 10.1093/database/baab010 33693667 PMC7947570

[B64] Liénard C. Pintart A. Bomont P. (2024). Neuronal autophagy: regulations and implications in Health and disease. Cells 13, 103. 10.3390/cells13010103 38201307 PMC10778363

[B65] Liu L. Liu T. Xie G. Zhu X. Wang Y. (2022). Ubiquitin ligase TRIM32 promotes dendrite arborization by mediating degradation of the epigenetic factor CDYL. FASEB J. 36, e22087. 10.1096/fj.202100031RR 34888944

[B66] Lu D. C. Niu T. Alaynick W. A. (2015). Molecular and cellular development of spinal cord locomotor circuitry. Front. Mol. Neurosci. 8, 25. 10.3389/fnmol.2015.00025 26136656 PMC4468382

[B67] Lubs H. A. Stevenson R. E. Schwartz C. E. (2012). Fragile X and X-linked intellectual disability: four decades of discovery. Am. J. Hum. Genet. 90, 579–590. 10.1016/j.ajhg.2012.02.018 22482801 PMC3322227

[B68] Ma P. Mao B. (2022). The many faces of the E3 ubiquitin ligase, RNF220, in neural development and beyond. Dev. Growth Differ. 64, 98–105. 10.1111/dgd.12756 34716995

[B69] Ma P. Song N.-N. Li Y. Zhang Q. Zhang L. Zhang L. (2019). Fine-tuning of shh/gli signaling gradient by non-proteolytic ubiquitination during neural patterning. Cell Rep. 28, 541–553.e4. 10.1016/j.celrep.2019.06.017 31291587

[B70] Mabb A. M. (2021). Historical perspective and progress on protein ubiquitination at glutamatergic synapses. Neuropharmacology 196, 108690. 10.1016/j.neuropharm.2021.108690 34197891 PMC8831078

[B71] Mabb A. M. Ehlers M. D. (2018). Arc ubiquitination in synaptic plasticity. Seminars Cell and Dev. Biol. 77, 10–16. 10.1016/j.semcdb.2017.09.009 28890418

[B72] Mabbitt P. D. Loreto A. Déry M.-A. Fletcher A. J. Stanley M. Pao K.-C. (2020). Structural basis for RING-Cys-Relay E3 ligase activity and its role in axon integrity. Nat. Chem. Biol. 16, 1227–1236. 10.1038/s41589-020-0598-6 32747811 PMC7610530

[B73] Maday S. Wallace K. E. Holzbaur E. L. F. (2012). Autophagosomes initiate distally and mature during transport toward the cell soma in primary neurons. J. Cell Biol. 196, 407–417. 10.1083/jcb.201106120 22331844 PMC3283992

[B74] Madiraju C. Novack J. P. Reed J. C. Matsuzawa S. (2022). K63 ubiquitination in immune signaling. Trends Immunol. 43, 148–162. 10.1016/j.it.2021.12.005 35033428 PMC8755460

[B75] Maenner M. J. Warren Z. Williams A. R. Amoakohene E. Bakian A. V. Bilder D. A. (2023). Prevalence and characteristics of autism Spectrum disorder among children aged 8 Years — autism and developmental disabilities monitoring network, 11 sites, United States, 2020. MMWR Surveill. Summ. 72, 1–14. 10.15585/mmwr.ss7202a1 PMC10042614 36952288

[B76] Margolis S. S. Sell G. L. Zbinden M. A. Bird L. M. (2015). Angelman syndrome. Neurotherapeutics 12, 641–650. 10.1007/s13311-015-0361-y 26040994 PMC4489961

[B77] Marin Navarro A. Pronk R. J. Van Der Geest A. T. Oliynyk G. Nordgren A. Arsenian-Henriksson M. (2020). p53 controls genomic stability and temporal differentiation of human neural stem cells and affects neural organization in human brain organoids. Cell Death Dis. 11, 52. 10.1038/s41419-019-2208-7 31974372 PMC6978389

[B78] Masui S. Nakatake Y. Toyooka Y. Shimosato D. Yagi R. Takahashi K. (2007). Pluripotency governed by Sox2 via regulation of Oct3/4 expression in mouse embryonic stem cells. Nat. Cell Biol. 9, 625–635. 10.1038/ncb1589 17515932

[B79] Matsumoto N. Leventer R. Kuc J. Mewborn S. Dudlicek L. L. Ramocki M. B. (2001). Mutation analysis of the DCX gene and genotype/phenotype correlation in subcortical band heterotopia. Eur. J. Hum. Genet. 9, 5–12. 10.1038/sj.ejhg.5200548 11175293

[B80] Matuskey D. Yang Y. Naganawa M. Koohsari S. Toyonaga T. Gravel P. (2024). 11C-UCB-J PET imaging is consistent with lower synaptic density in autistic adults. Mol. Psychiatry 30, 1610–1616. 10.1038/s41380-024-02776-2 39367053

[B81] McClellan A. J. Laugesen S. H. Ellgaard L. (2019). Cellular functions and molecular mechanisms of non-lysine ubiquitination. Open Biol. 9, 190147. 10.1098/rsob.190147 31530095 PMC6769291

[B82] Menon S. Boyer N. P. Winkle C. C. McClain L. M. Hanlin C. C. Pandey D. (2015). The E3 ubiquitin ligase TRIM9 is a filopodia off switch required for netrin-dependent axon guidance. Dev. Cell 35, 698–712. 10.1016/j.devcel.2015.11.022 26702829 PMC4707677

[B83] Metzger M. B. Pruneda J. N. Klevit R. E. Weissman A. M. (2014). RING-type E3 ligases: master manipulators of E2 ubiquitin-conjugating enzymes and ubiquitination. Biochim. Biophys. Acta 1843, 47–60. 10.1016/j.bbamcr.2013.05.026 23747565 PMC4109693

[B84] Mizushima N. (2024). Ubiquitin in autophagy and non-protein ubiquitination. Nat. Struct. Mol. Biol. 31, 208–209. 10.1038/s41594-024-01217-6 38366228

[B85] Moore S. W. Tessier-Lavigne M. Kennedy T. E. (2007). “Netrins and their receptors,” in Axon growth and guidance. Editor Bagnard D. (New York, NY: Springer New York), 17–31. 10.1007/978-0-387-76715-4_2

[B86] Münch C. Dikic I. (2018). Hitchhiking on selective autophagy. Nat. Cell Biol. 20, 122–124. 10.1038/s41556-018-0036-0 29371706

[B87] Mund T. Pelham H. R. B. (2009). Control of the activity of WW-HECT domain E3 ubiquitin ligases by NDFIP proteins. EMBO Rep. 10, 501–507. 10.1038/embor.2009.30 19343052 PMC2680872

[B88] Musaus M. Navabpour S. Jarome T. J. (2020). The diversity of linkage-specific polyubiquitin chains and their role in synaptic plasticity and memory formation. Neurobiol. Learn Mem. 174, 107286. 10.1016/j.nlm.2020.107286 32745599 PMC7484030

[B89] Mutalik S. P. Ho C. T. O’Shaughnessy E. C. Frasineanu A. G. Shah A. B. Gupton S. L. (2025). TRIM9 controls growth cone responses to netrin through DCC and UNC5C. J. Neurochem. 169, e70002. 10.1111/jnc.70002 39871643 PMC11834693

[B90] Muthusamy B. Nguyen T. T. Bandari A. K. Basheer S. Selvan L. D. N. Chandel D. (2020). Exome sequencing reveals a novel splice site variant in HUWE1 gene in patients with suspected Say-Meyer syndrome. Eur. J. Med. Genet. 63, 103635. 10.1016/j.ejmg.2019.02.007 30797980 PMC6974397

[B91] Myat A. Henry P. McCabe V. Flintoft L. Rotin D. Tear G. (2002). Drosophila Nedd4, a ubiquitin ligase, is recruited by commissureless to control cell surface levels of the Roundabout receptor. Neuron 35, 447–459. 10.1016/S0896-6273(02)00795-X 12165468

[B92] Nishimura T. Tooze S. A. (2020). Emerging roles of ATG proteins and membrane lipids in autophagosome formation. Cell Discov. 6, 32. 10.1038/s41421-020-0161-3 32509328 PMC7248066

[B93] Obara K. Ohsumi Y. (2011). Atg14: a key player in orchestrating autophagy. Int. J. Cell Biol. 2011, 713435. 10.1155/2011/713435 22013444 PMC3195510

[B94] O’Donnell M. Chance R. K. Bashaw G. J. (2009). Axon growth and guidance: receptor regulation and signal transduction. Annu. Rev. Neurosci. 32, 383–412. 10.1146/annurev.neuro.051508.135614 19400716 PMC4765433

[B95] Otten E. G. Werner E. Crespillo-Casado A. Boyle K. B. Dharamdasani V. Pathe C. (2021). Ubiquitylation of lipopolysaccharide by RNF213 during bacterial infection. Nature 594, 111–116. 10.1038/s41586-021-03566-4 34012115 PMC7610904

[B96] Paik H. Oh C. Hussain S. Seo S. Park S. W. Ko T. L. (2024). ELiAH: the atlas of E3 ligases in human tissues for targeted protein degradation with reduced off-target effect. Database 2024, baae111. 10.1093/database/baae111 39395186 PMC11470751

[B97] Pao K.-C. Wood N. T. Knebel A. Rafie K. Stanley M. Mabbitt P. D. (2018). Activity-based E3 ligase profiling uncovers an E3 ligase with esterification activity. Nature 556, 381–385. 10.1038/s41586-018-0026-1 29643511

[B98] Parato J. Bartolini F. (2021). The microtubule cytoskeleton at the synapse. Neurosci. Lett. 753, 135850. 10.1016/j.neulet.2021.135850 33775740 PMC8089059

[B99] Persson M. Stamataki D. te Welscher P. Andersson E. Böse J. Rüther U. (2002). Dorsal-ventral patterning of the spinal cord requires Gli3 transcriptional repressor activity. Genes Dev. 16, 2865–2878. 10.1101/gad.243402 12435629 PMC187477

[B100] Plooster M. Menon S. Winkle C. C. Urbina F. L. Monkiewicz C. Phend K. D. (2017). TRIM9-dependent ubiquitination of DCC constrains kinase signaling, exocytosis, and axon branching. MBoC 28, 2374–2385. 10.1091/mbc.e16-08-0594 28701345 PMC5576901

[B101] Qi C. Liu S. Qin R. Zhang Y. Wang G. Shang Y. (2014). Coordinated regulation of dendrite arborization by epigenetic factors CDYL and EZH2. J. Neurosci. 34, 4494–4508. 10.1523/JNEUROSCI.3647-13.2014 24671995 PMC6608133

[B102] Ravanelli A. M. Appel B. (2015). Motor neurons and oligodendrocytes arise from distinct cell lineages by progenitor recruitment. Genes Dev. 29, 2504–2515. 10.1101/gad.271312.115 26584621 PMC4691953

[B103] Ren X. Ming G. Xie Y. Hong Y. Sun D. Zhao Z. (2004). Focal adhesion kinase in netrin-1 signaling. Nat. Neurosci. 7, 1204–1212. 10.1038/nn1330 15494733

[B104] Roy B. Amemasor E. Hussain S. Castro K. (2023). UBE3A: the role in autism Spectrum disorders (ASDs) and a potential candidate for biomarker studies and designing therapeutic strategies. Diseases 12, 7. 10.3390/diseases12010007 38248358 PMC10814747

[B105] Ruiz i Altaba A. (1998). Combinatorial Gli gene function in floor plate and neuronal inductions by Sonic hedgehog. Development 125, 2203–2212. 10.1242/dev.125.12.2203 9584120

[B106] Schulman B. A. Harper J. W. (2009). Ubiquitin-like protein activation by E1 enzymes: the apex for downstream signalling pathways. Nat. Rev. Mol. Cell Biol. 10, 319–331. 10.1038/nrm2673 19352404 PMC2712597

[B107] Scott F. L. Denault J.-B. Riedl S. J. Shin H. Renatus M. Salvesen G. S. (2005). XIAP inhibits caspase-3 and -7 using two binding sites: evolutionarily conserved mechanism of IAPs. EMBO J. 24, 645–655. 10.1038/sj.emboj.7600544 15650747 PMC548652

[B108] Shim T. Kim J. Y. Kim W. Lee Y.-I. Cho B. Moon C. (2024). Cullin-RING E3 ubiquitin ligase 4 regulates neurite morphogenesis during neurodevelopment. iScience 27, 108933. 10.1016/j.isci.2024.108933 38318354 PMC10839267

[B109] Smith S. E. P. Zhou Y.-D. Zhang G. Jin Z. Stoppel D. C. Anderson M. P. (2011). Increased gene dosage of Ube3a results in autism traits and decreased glutamate synaptic transmission in mice. Sci. Transl. Med. 3, 103ra97. 10.1126/scitranslmed.3002627 PMC3356696 21974935

[B110] Song J. Merrill R. A. Usachev A. Y. Strack S. (2021). The X-linked intellectual disability gene product and E3 ubiquitin ligase KLHL15 degrades doublecortin proteins to constrain neuronal dendritogenesis. J. Biol. Chem. 296, 100082. 10.1074/jbc.RA120.016210 33199366 PMC7948412

[B111] Srivatsa S. Parthasarathy S. Britanova O. Bormuth I. Donahoo A.-L. Ackerman S. L. (2014). Unc5C and DCC act downstream of Ctip2 and Satb2 and contribute to corpus callosum formation. Nat. Commun. 5, 3708. 10.1038/ncomms4708 24739528 PMC3997811

[B112] Stevenson R. E. Holden K. R. Rogers R. C. Schwartz C. E. (2012). Seizures and X-linked intellectual disability. Eur. J. Med. Genet. 55, 307–312. 10.1016/j.ejmg.2012.01.017 22377486 PMC3531238

[B113] Stewart M. D. Ritterhoff T. Klevit R. E. Brzovic P. S. (2016). E2 enzymes: more than just middle men. Cell Res. 26, 423–440. 10.1038/cr.2016.35 27002219 PMC4822130

[B114] Sullivan K. G. Bashaw G. J. (2024). Commissureless acts as a substrate adapter in a conserved Nedd4 E3 ubiquitin ligase pathway to promote axon growth across the midline. bioRxiv. 10.1101/2023.10.13.562283 PMC12101832 40407164

[B115] Suryadinata R. Roesley S. N. A. Yang G. Sarčević B. (2014). Mechanisms of generating polyubiquitin chains of different topology. Cells 3, 674–689. 10.3390/cells3030674 24987835 PMC4197637

[B116] Swatek K. N. Komander D. (2016). Ubiquitin modifications. Cell Res. 26, 399–422. 10.1038/cr.2016.39 27012465 PMC4822133

[B117] Telias M. Ben-Yosef D. (2014). Modeling neurodevelopmental disorders using human pluripotent stem cells. Stem Cell Rev Rep 10, 494–511. 10.1007/s12015-014-9507-2 24728983

[B118] Terawaki S. Camosseto V. Prete F. Wenger T. Papadopoulos A. Rondeau C. (2015). RUN and FYVE domain-containing protein 4 enhances autophagy and lysosome tethering in response to Interleukin-4. J. Cell Biol. 210, 1133–1152. 10.1083/jcb.201501059 26416964 PMC4586740

[B119] Wang F. Bach I. (2019). Rlim/Rnf12, Rex1, and X chromosome inactivation. Front. Cell Dev. Biol. 7, 258. 10.3389/fcell.2019.00258 31737626 PMC6834644

[B120] Wang M. Luo W. Zhang Y. Yang R. Li X. Guo Y. (2020a). Trim32 suppresses cerebellar development and tumorigenesis by degrading Gli1/sonic hedgehog signaling. Cell Death Differ. 27, 1286–1299. 10.1038/s41418-019-0415-5 31527798 PMC7206143

[B121] Wang T. Wang J. Wang J. Mao L. Tang B. Vanderklish P. W. (2019a). HAP1 is an *in vivo* UBE3A target that augments autophagy in a mouse model of Angelman syndrome. Neurobiol. Dis. 132, 104585. 10.1016/j.nbd.2019.104585 31445164

[B122] Wang X. S. Cotton T. R. Trevelyan S. J. Richardson L. W. Lee W. T. Silke J. (2023). The unifying catalytic mechanism of the RING-between-RING E3 ubiquitin ligase family. Nat. Commun. 14, 168. 10.1038/s41467-023-35871-z 36631489 PMC9834252

[B123] Wang Y. Argiles-Castillo D. Kane E. I. Zhou A. Spratt D. E. (2020b). HECT E3 ubiquitin ligases - emerging insights into their biological roles and disease relevance. J. Cell Sci. 133, jcs228072. 10.1242/jcs.228072 32265230 PMC7157599

[B124] Wang Y.-B. Song N.-N. Zhang L. Ma P. Chen J.-Y. Huang Y. (2022). Rnf220 is implicated in the dorsoventral patterning of the hindbrain neural tube in mice. Front. Cell Dev. Biol. 10, 831365. 10.3389/fcell.2022.831365 35399523 PMC8988044

[B125] Wang Z. Liu Z. Chen X. Li J. Yao W. Huang S. (2019b). A multi-lock inhibitory mechanism for fine-tuning enzyme activities of the HECT family E3 ligases. Nat. Commun. 10, 3162. 10.1038/s41467-019-11224-7 31320636 PMC6639328

[B126] Weissman A. M. (2001). Themes and variations on ubiquitylation. Nat. Rev. Mol. Cell Biol. 2, 169–178. 10.1038/35056563 11265246

[B127] Widagdo J. Guntupalli S. Jang S. E. Anggono V. (2017). Regulation of AMPA receptor trafficking by protein ubiquitination. Front. Mol. Neurosci. 10, 347. 10.3389/fnmol.2017.00347 29123470 PMC5662755

[B128] Wong Y. C. Holzbaur E. L. F. (2014). The regulation of autophagosome dynamics by Huntingtin and HAP1 is disrupted by expression of mutant Huntingtin, leading to defective cargo degradation. J. Neurosci. 34, 1293–1305. 10.1523/JNEUROSCI.1870-13.2014 24453320 PMC3898289

[B129] Wu Z. Makihara S. Yam P. T. Teo S. Renier N. Balekoglu N. (2019). Long-range guidance of spinal commissural axons by Netrin1 and sonic hedgehog from midline floor plate cells. Neuron 101, 635–647.e4. 10.1016/j.neuron.2018.12.025 30661738

[B130] Yang D. Cheng D. Tu Q. Yang H. Sun B. Yan L. (2018). HUWE1 controls the development of non-small cell lung cancer through down-regulation of p53. Theranostics 8, 3517–3529. 10.7150/thno.24401 30026863 PMC6037029

[B131] Yi J. J. Berrios J. Newbern J. M. Snider W. D. Philpot B. D. Hahn K. M. (2015). An autism-linked mutation disables phosphorylation control of UBE3A. Cell 162, 795–807. 10.1016/j.cell.2015.06.045 26255772 PMC4537845

[B132] Zang Y. Chaudhari K. Bashaw G. J. (2021). New insights into the molecular mechanisms of axon guidance receptor regulation and signaling. Curr. Top. Dev. Biol. 142, 147–196. 10.1016/bs.ctdb.2020.11.008 33706917 PMC8456978

[B133] Zhang L. Qin Y. Wu G. Wang J. Cao J. Wang Y. (2020). PRRG4 promotes breast cancer metastasis through the recruitment of NEDD4 and downregulation of Robo1. Oncogene 39, 7196–7208. 10.1038/s41388-020-01494-7 33037408

[B134] Zhang Y. Yang X. Gui B. Xie G. Zhang D. Shang Y. (2011). Corepressor protein CDYL functions as a molecular bridge between Polycomb repressor complex 2 and repressive chromatin mark trimethylated histone lysine 27. J. Biol. Chem. 286, 42414–42425. 10.1074/jbc.M111.271064 22009739 PMC3234934

[B135] Zhao X. Heng J. I.-T. Guardavaccaro D. Jiang R. Pagano M. Guillemot F. (2008). The HECT-domain ubiquitin ligase Huwe1 controls neural differentiation and proliferation by destabilizing the N-Myc oncoprotein. Nat. Cell Biol. 10, 643–653. 10.1038/ncb1727 18488021 PMC2680438

[B136] Zheng N. Shabek N. (2017). Ubiquitin ligases: structure, function, and regulation. Annu. Rev. Biochem. 86, 129–157. 10.1146/annurev-biochem-060815-014922 28375744

[B137] Zhu J.-W. Jia W.-Q. Zhou H. Li Y.-F. Zou M.-M. Wang Z.-T. (2021). Deficiency of TRIM32 impairs motor function and Purkinje cells in mid-aged mice. Front. Aging Neurosci. 13, 697494. 10.3389/fnagi.2021.697494 34421574 PMC8377415

[B138] Zinngrebe J. Montinaro A. Peltzer N. Walczak H. (2014). Ubiquitin in the immune system. EMBO Rep. 15, 322. 10.1002/embr.201470030 PMC4303447 24375678

[B139] Zou Y. Liu Q. Chen B. Zhang X. Guo C. Zhou H. (2007). Mutation in CUL4B, which encodes a member of cullin-RING ubiquitin ligase complex, causes X-linked mental retardation. Am. J. Hum. Genet. 80, 561–566. 10.1086/512489 17273978 PMC1821105

